# Consequences of the COVID-19 Pandemic on the Mental Health of Patients in Opioid Substitution Treatment

**DOI:** 10.1192/j.eurpsy.2022.958

**Published:** 2022-09-01

**Authors:** J. Rosenleitner, I. Fuchs-Leitner, N. Gerstgrasser, K. Yazdi

**Affiliations:** Kepler University Hospital Linz, Department Of Psychiatry - Specialization Addiction Medicine, Linz, Austria

**Keywords:** DAS-21, Covid-19, Opioid Substitution Treatment (OST), PTSD

## Abstract

**Introduction:**

The many negative consequences of the COVID-19 pandemic especially on vulnerable groups like patients suffering from drug addiction have been anticipated by experts early on. While drug consumption patterns of patients in opioid substitution treatment (OST, N=24) seemed hardly influenced at the early stage of the pandemic in Austria, the impact on the mental health of this population remained unclear.

**Objectives:**

The main goal was to investigate long-term consequences of the pandemic in terms of PTSD and clinical symptoms of depression, anxiety and stress among patients in OST between December 2020 and February 2021.

**Methods:**

In a cross-sectional survey study (N=123) an adapted version of the impact of event scale (IES-R) was applied to evaluate PTSD symptoms due to the COVID-19 pandemic. Clinical symptoms were assessed by the depression, anxiety and stress scale (DASS-21), and respective changes due to the pandemic were documented. Sociodemographic and COVID-19 related factors, as well as data on drug consumption patterns were collected.

**Results:**

A binary logistic regression analysis confirmed the negative long-term consequences of psychological and economic COVID-19 related factors on a higher risk for PTSD due to the pandemic. The high-risk PTSD group also demonstrated higher levels and a deterioration of depression, anxiety and stress symptoms since the pandemic.

**Conclusions:**

Among our sample of patients in OST, 27% were at risk of PTSD due to the pandemic, and 30 to 50% reported concerning levels of depression, anxiety and stress. Health care facilities might use these findings as a valuable source of information, when special attention is needed.

**Disclosure:**

No significant relationships.

